# Comparative Genomics Reveals a Well-Conserved Intrinsic Resistome in the Emerging Multidrug-Resistant Pathogen Cupriavidus gilardii

**DOI:** 10.1128/mSphere.00631-19

**Published:** 2019-10-02

**Authors:** Cristian Ruiz, Ashley McCarley, Manuel Luis Espejo, Kerry K. Cooper, Dana E. Harmon

**Affiliations:** a Department of Biology, California State University, Northridge, Northridge, California, USA; b School of Animal and Comparative Biomedical Sciences, University of Arizona, Tucson, Arizona, USA; JMI Laboratories

**Keywords:** *Cupriavidus*, *Cupriavidus gilardii*, antibiotic resistance, carbapenems, aminoglycosides

## Abstract

The Gram-negative bacterium Cupriavidus gilardii is an emerging multidrug-resistant pathogen found in many environments. However, little is known about this species or its antibiotic resistance mechanisms. We used biochemical tests, antibiotic susceptibility experiments, and whole-genome sequencing to characterize an environmental C. gilardii isolate. Like clinical isolates, this isolate was resistant to meropenem, gentamicin, and other antibiotics. Resistance to these antibiotics appeared to be related to the large number of intrinsic antibiotic resistance genes found in this isolate. As determined by comparative genomics, this resistome was also well conserved in the only two other C. gilardii strains sequenced to date. The intrinsic resistome of C. gilardii did not include the colistin resistance gene *mcr-5*, which was in a transposon present only in one strain. The intrinsic resistome of C. gilardii was comprised of (i) many multidrug efflux pumps, such as a homolog of the Pseudomonas aeruginosa MexAB-OprM pump that may be involved in resistance to meropenem, other β-lactams, and aminoglycosides; (ii) a novel β-lactamase (OXA-837) that decreases susceptibility to ampicillin but not to other β-lactams tested; (iii) a new aminoglycoside 3-*N*-acetyltransferase [AAC(3)-IVb, AacC10] that decreases susceptibility to gentamicin and tobramycin; and (iv) a novel partially conserved aminoglycoside 3ʺ-adenylyltransferase [ANT(3ʺ)-Ib, AadA32] that decreases susceptibility to spectinomycin and streptomycin. These findings provide the first mechanistic insight into the intrinsic resistance of C. gilardii to multiple antibiotics and its ability to become resistant to an increasing number of drugs during therapy.

**IMPORTANCE** Cupriavidus gilardii is a bacterium that is gaining increasing attention both as an infectious agent and because of its potential use in the detoxification of toxic compounds and other biotechnological applications. In recent years, however, there has been an increasing number of reported infections, some of them fatal, caused by C. gilardii. These infections are hard to treat because this bacterium is naturally resistant to many antibiotics, including last-resort antibiotics, such as carbapenems. Moreover, this bacterium often becomes resistant to additional antibiotics during therapy. However, little is known about C. gilardii and its antibiotic resistance mechanisms. The significance of our research is in providing, for the first time, whole-genome information about the natural antibiotic resistance genes found in this bacterium and their conservation among different C. gilardii strains. This information may provide new insights into the appropriate use of antibiotics in combating infections caused by this emerging pathogen.

## Abstract (precis)

Cupriavidus gilardii is a bacterium that is gaining increasing attention both as an infectious agent and because of its potential use in the detoxification of toxic compounds and other biotechnological applications. In recent years, however, there has been an increasing number of reported infections, some of them fatal, caused by C. gilardii. These infections are hard to treat because this bacterium is naturally resistant to many antibiotics, including last-resort antibiotics, such as carbapenems. Moreover, this bacterium often becomes resistant to additional antibiotics during therapy. However, little is known about C. gilardii and its antibiotic resistance mechanisms. The significance of our research is in providing, for the first time, whole-genome information about the natural antibiotic resistance genes found in this bacterium and their conservation among different C. gilardii strains. This information may provide new insights into the appropriate use of antibiotics in combating infections caused by this emerging pathogen.

## INTRODUCTION

*Cupriavidus* is a genus of Gram-negative, rod-shaped, motile, glucose-nonfermenting bacteria belonging to the *Betaproteobacteria* class and the *Burkholderiaceae* family (1, 2). This genus has a complex taxonomic history, including the previous classification of isolates as *Ralstonia* and *Wautersia* species (1, 2). *Cupriavidus* species have been isolated from both environmental and clinical samples and are generally highly resistant to copper and other metals (1–3).

Cupriavidus gilardii, formerly known as Ralstonia gilardii and Wautersia gilardii, was first identified in 1999 by Coenye et al. (4) by studying Alcaligenes faecalis-like environmental and clinical isolates. Since then, C. gilardii has been isolated from multiple ecological niches, including untreated drinking water (5), urban pond water (6), agricultural soil (7), soil contaminated with heavy metals (8), soil containing natural asphalt (9), plants (10), and human clinical samples (11–17). At the environmental and biotechnological levels, this organism has gained attention because of its potential role as an indicator of heavy metal contamination (8), as well as its ability to degrade herbicides (7) and other toxic hydrocarbons, such as naphthenic acids (9).

At the clinical level, C. gilardii has been isolated from multiple human samples of cerebrospinal fluid, bone marrow, wounds, furuncles, and the respiratory tract, and from respiratory secretions of cystic fibrosis patients (4, 12). Because the pathogenicity associated with the presence of C. gilardii in these clinical samples was not studied and because it is difficult to accurately identify C. gilardii by the standard methods used in hospitals, infections caused by this organism have been underdiagnosed (14–16). However, recent reports of infections caused by C. gilardii suggest that this organism may be an emerging pathogen, especially in immunocompromised or elderly patients (11, 13–17). Such pathogenicity, its innate resistance to multiple antibiotics (including last-resort antibiotics, such as carbapenems), and its ability to acquire new resistances as it colonizes its human host make C. gilardii an increasing health concern (11, 13–17).

Further studies are necessary to better characterize C. gilardii and identify which strains may be applied in biotechnology and which strains may cause disease in humans. It is essential to determine which genes allow different strains to thrive in different environments, as well as to identify which genes contribute to antibiotic resistance in this species. However, only two genomes of this organism have been reported so far. The first to be reported is the complete genome of C. gilardii CR3 (9), which has 2 chromosomes (GenBank accession numbers CP010516 and CP010517). Because this strain was isolated from natural asphalt-containing soil, can degrade naphthenic acids, and resists multiple heavy metals, it has been proposed to be an attractive bioremediation agent for petroleum-polluted environments (9). The second one to be reported is the incompletely assembled draft genome of C. gilardii JZ4 (GenBank accession number LVXY00000000). This strain shows growth-promoting effects and was isolated from the roots of a desert plant (10). Here, we report the characterization and genome of a C. gilardii strain previously isolated from surface water from an urban pond in Los Angeles, CA (6), as part of a wider effort to isolate environmental antibiotic-resistant bacteria. Genomic, phylogenetic, and comparative genomic analyses of our water isolate and previous soil and plant isolates provide important insights into the core resistome of C. gilardii, especially, about the intrinsic β-lactam and aminoglycoside resistance mechanisms found in this species.

## RESULTS AND DISCUSSION

### Description and characterization of C. gilardii W2-2.

C. gilardii strain W2-2 was isolated from the surface water of an artificial urban pond located on the California State University, Northridge, campus in Los Angeles, CA, using MacConkey agar plates supplemented with meropenem and incubated under aerobic conditions for 24 h at 37°C (6). This isolate was identified to be C. gilardii by 16S rRNA gene sequencing and was initially characterized to be meropenem and gentamicin resistant, imipenem intermediate or susceptible (according to the breakpoints for *Enterobacteriaceae* and Pseudomonas aeruginosa, respectively), and cefotaxime, ciprofloxacin, and tetracycline susceptible (6). This antibiotic susceptibility profile, especially resistance to meropenem and gentamicin, is very similar to the susceptibility profile of the C. gilardii clinical isolates described to date (11, 13–17). Because of the overall little knowledge about C. gilardii, especially regarding its antibiotic resistance mechanisms, this isolate was selected for further investigation. Phenotypic characterization of strain W2-2 revealed that its cell morphology and biochemical profile are identical to those of C. gilardii LMG 5886^T^ (ATCC 700815, DSM 17292) (2, 4) (see Table S1 in the supplemental material).

10.1128/mSphere.00631-19.2 TABLE S1 Phenotypic characterization of C. gilardii W2-2. Download Table S1, PDF file, 0.08 MB. Copyright © 2019 Ruiz et al. 2019 Ruiz et al. This content is distributed under the terms of the Creative Commons Attribution 4.0 International license.

### General genome structure and features of C. gilardii W2-2.

**FIG 1:**
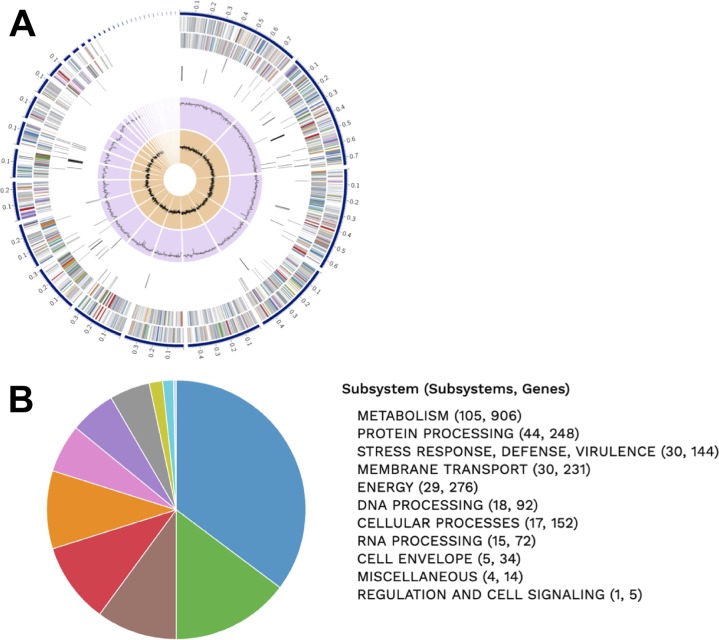
(A) Circular graphical display of the distribution of the genome annotations of C. gilardii strain W2-2, which includes, from the outer to the inner rings, the contigs (the scale is in mega-base pairs, ordered by decreasing size), coding DNA sequences (CDS) on the forward strand, CDS on the reverse strand, RNA genes, CDS with homology to known antimicrobial resistance genes, CDS with homology to know virulence factors, and the GC content and GC skew (G − C/G + C ratio). The colors of the CDS on the forward and reverse strands indicate the subsystem that these genes belong to (see panel B for additional details). (B) Graphical representation of the major functional categories and subsystems (specific biological processes or structural complexes) in which the annotated genes of C. gilardii W2-2 are involved. For each major functional category, the number of subsystems (numbers on the left) and the total number of annotated genes (numbers on the right) are shown in parentheses.

We sequenced and analyzed the genome of C. gilardii W2-2 using the comprehensive genome analysis service at the Pathosystems Resource Integration Center (PATRIC). This genome was assembled into 38 contigs (Table 1). Based on the obtained *L*_50_ and *N*_50_ values and based on a comparison to the two previously sequenced C. gilardii genomes—the complete genome of strain CR3 (9) (GenBank accession numbers CP010516 and CP010517 for chromosomes 1 and 2, respectively) and the not fully assembled draft genome of strain JZ4 (GenBank accession number LVXY00000000)—the draft genome of strain W2-2 covers both C. gilardii chromosomes and is of good quality. Full details about the genome features of strain W2-2 are provided in Tables 1 and 2 and Fig. 1. This initial analysis also revealed that strain W2-2 has multiple genes predicted to be involved in antibiotic resistance (40 genes) or virulence (11 genes) (Fig. 1; Table 1).

**TABLE 1:** General features of the genome of C. gilardii W2-2, determined using the PATRIC comprehensive genome analysis service

Feature	Value for C. gilardii W2-2
Size (bp)	5,595,578
GC content (%)	67.94
No. of contigs	38
Contig *L*_50_ value	5
Contig *N*_50_ value	475, 905
No. of tRNAs	54
No. of rRNAs	4
Total no. of coding DNA sequences	5,073
No. of hypothetical proteins	1,189
No. of proteins with:	
Functional assignments	3,884
EC number assignments	1,158
GO assignments	991
Pathway assignments	883
Genus-specific family assignments (PLfam)	4,430
Cross-genus family assignments (PGfam)	4,530
No. of predicted genes involved in:	
Antibiotic resistance	40 (30)^*a*^
Virulence	11

a The initial genome analysis using the comprehensive genome analysis service at PATRIC revealed 40 genes that mapped to CARD or the PATRIC AMR database. Further analysis of these genes and of the rest of the annotated genes in each genome revealed a total of 30 genes strongly predicted to be involved in antibiotic resistance because of the similarity of their predicted proteins to one or more antibiotic resistance proteins in CARD (see Table S2 in the supplemental material).

**TABLE 2:** Genome structure comparison for currently known C. gilardii genomes determined with both the PATRIC comprehensive genome analysis service and the latest GenBank genome update

Strain	Strain isolation source	Status	Genome size (Mbp)	GC content (%)	No. of chromosomes/ no. of plasmids	No. of^*a*^:					Latest update in GenBank (yr/day/mo) or source
						CDS	rRNA	tRNA	Other RNAs	Total genes	
CR3^*b*^	Soil with natural asphalt (CA, USA)	Complete	5.58	67.55	2/0	5,412 (4,401)	12	59	0 (3)	5,483 (4,992)	2017/04/11
JZ4^*c*^	Plant root endophyte (Jizan, Saudi Arabia)	Draft	5.56	67.70		5,042 (4,772)	9 (10)	53	0 (3)	5,104 (4,889)	2017/04/12
W2-2	Surface pond water (CA, USA)	Draft	5.60	67.94		5,073 (4,848)	4	54 (53)	0 (3)	5,131 (4,908)	This study

a When the values for the numbers of coding DNA sequences (CDS), rRNAs, tRNAs, other RNAs, or total genes were different between the PATRIC service and the GenBank database, the value from PATRIC is shown and the value from GenBank is shown in parentheses. In general, these differences occurred mostly for coding DNA sequences, and, thus, total genes and are related to the different scoring of pseudogenes (Rebecca Wattam, University of Virginia, personal communication).

b C. gilardii CR3 (9) (GenBank accession numbers CP010516 and CP010517).

c C. gilardii JZ4 (10) (GenBank accession number LVXY00000000).

10.1128/mSphere.00631-19.3 TABLE S2 Antibiotic resistance determinants found in C. gilardii W2-2 with a strong match in CARD. Download Table S2, PDF file, 0.1 MB. Copyright © 2019 Ruiz et al. 2019 Ruiz et al. This content is distributed under the terms of the Creative Commons Attribution 4.0 International license.

### Whole-genome phylogenetic analysis of the genus *Cupriavidus* and comparative genomic analysis of C. gilardii genomes.

**FIG 2:**
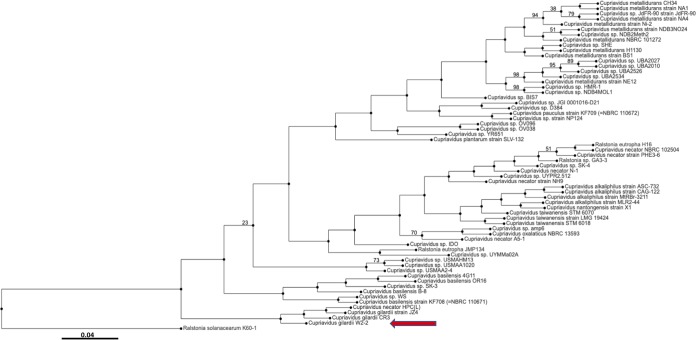
Whole-genome phylogenetic tree of the genus *Cupriavidus* constructed using the RAxML algorithm and the progressive refinement method. The tree is based on the 61 complete or draft *Cupriavidus* genomes currently available in the PATRIC database and the C. gilardii genome obtained in this study. The tree was rooted using the genome of the type strain Ralstonia solanacearum K60-1 as the outgroup. Only branch support values of <100% are shown. The scale bar represents the number of substitutions per site.

Whole-genome phylogenetic analysis of C. gilardii W2-2 and the other 61 *Cupriavidus* genomes currently available in the PATRIC database confirmed that this isolate is clearly a member of the genus *Cupriavidu*s and is in a monophyletic group with the other two C. gilardii strains sequenced so far (Fig. 2). This analysis also revealed that Cupriavidus necator HPC(L), which was originally described as a *Cupriavidus* sp. and then labeled as C. necator before the first C. gilardii genomes were sequenced, should probably be reclassified as C. gilardii (Fig. 2). Interestingly, C. gilardii strain CR3 (isolated from soil) was found to be more closely related to strain JZ4 (isolated from plant roots) than to our water isolate (Fig. 2). Finally, our analysis suggests that C. gilardii diverged earlier in the genus history than other species. For these other species, many taxonomical groups, such as the C. basiliensis, C. alkaliphilus/C. necator/C. taiwanensis, and C. metallidurans groups, appear to be mostly well-defined, although some isolates, such as C. metallidurans NE12, may need to be reclassified (Fig. 2).

A more in-depth comparative genome analysis of C. gilardii W2-2 and the other two currently available C. gilardii genomes of strains CR3 and JZ4 revealed that all three C. gilardii strains have a very similar genome size and GC content (Table 2). Similarly, synteny analysis revealed that the genomes of all C. gilardii strains analyzed are mostly composed of well-conserved sequence blocks (Fig. S1). However, compared to the reference strain, CR3, we also found an extensive rearrangement of these sequence blocks in our W2-2 isolate and, to a lesser degree, in strain JZ4 (Fig. S1).

10.1128/mSphere.00631-19.1 FIG S1 Synteny plot analysis of C. gilardii genomes from strains W2-2 (this study; top), CR3 (GenBank accession numbers CP010516 to CP010517; selected as a reference; center) and JZ4 (GenBank accession number LVXY00000000; bottom). Download FIG S1, TIF file, 2.8 MB. Copyright © 2019 Ruiz et al. 2019 Ruiz et al. This content is distributed under the terms of the Creative Commons Attribution 4.0 International license.

**FIG 3:**
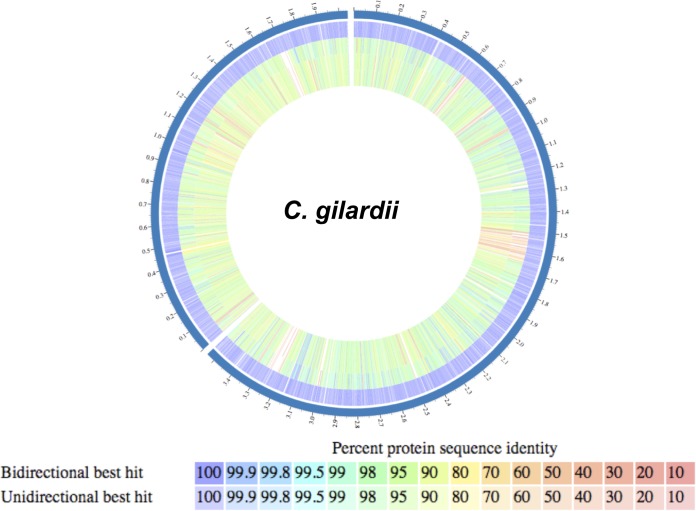
Whole-genome comparative proteomics schematic display of currently available genomes identified as C. gilardii. The tracks, from outside to inside, are (i) strain CR3 chromosomes 1 and 2 (dark blue track, the numbers represent mega-base pairs; GenBank accession numbers CP010516 to CP010517); (ii) the strain CR3 proteome, which was selected as the reference; (iii) the strain JZ4 (GenBank accession number LVXY00000000) proteome; and (iv) the W2-2 proteome (this study). For strains JZ4 and W2-2, each predicted protein-coding gene is marked as either unique, a unidirectional best hit, or a bidirectional best hit and is color coded according to their BLASTP percent similarity compared to strain CR3.

Comparative analysis of the predicted proteome of all 3 C. gilardii strains confirmed a large degree of conservation (85% to 99% identity for most genes) within this species (Fig. 3). Genes found to be unique to strain CR3 compared to the other C. gilardii strains were mostly phage-related, capsule biosynthesis, and hypothetical protein genes. Most other genes found in strain CR3 were also found to be present and highly conserved in strain JZ4 (94% average identity compared to strain CR3) and our water isolate (92% average identity compared to strain CR3) (Fig. 3). As with our previous analysis, the higher identity between strains CR3 and JZ4 than between strains CR3 and W2-2 seems to support the hypothesis about how the type of environment plays a greater role in the evolution of this species than geographical differences.

### Analysis of the antibiotic resistance genes of C. gilardii W2-2 and comparison with other C. gilardii strains reveals a large and complex resistome within this species.

Two of the major concerns about C. gilardii as an emerging pathogen are its innate resistance to multiple antibiotics and its ability to acquire new resistances during therapy, often requiring a combination of multiple drugs and changes in therapy during the treatment of patients (11, 13–17). However, no C. gilardii clinical isolate has been sequenced to date, nor is there any knowledge about the antibiotic resistance mechanisms found in this species, except for a recent report of a transposon containing the *mcr-5* colistin resistance gene identified first in a Salmonella enterica serovar Paratyphi plasmid and then, using BLAST analysis, in chromosome 1 of C. gilardii CR3 (18). Therefore, we studied in more detail the antibiotic resistance in our C. gilardii water isolate, further analyzed its genome to identify its antibiotic resistance determinants, and used comparative genomic studies to determine how extended and conserved these determinants are within C. gilardii.

Our initial characterization revealed that our water isolate is resistant to meropenem and gentamicin (6). Resistance to β-lactams (especially meropenem) and aminoglycosides (especially gentamicin and tobramycin) is one of the major characteristics of C. gilardii clinical isolates (11, 13–17). Thus, we further studied susceptibility to these two groups of antibiotics in our isolate. We found that C. gilardii W2-2 is extensively resistant to both groups of antibiotics (Table 3). For β-lactams, strain W2-2 was completely resistant (inhibition zone diameter, 0 mm) to the carbapenems meropenem and ertapenem, as well as to two other β-lactams, ampicillin and amoxicillin-clavulanate, whereas it was susceptible to imipenem and cefotaxime (Table 3). For aminoglycosides, this isolate was resistant to gentamicin, tobramycin, and streptomycin; intermediate to spectinomycin; and sensitive to amikacin and kanamycin (Table 3). These findings are remarkably consistent with the susceptibility profile for these two classes of antibiotics reported for C. gilardii clinical isolates (11, 13–17), which suggests that resistance to β-lactams and aminoglycosides in C. gilardii mostly occurs by intrinsic mechanisms common to both clinical and environmental isolates. To identify these mechanisms, we investigated the genome of our isolate and compared it to the two other available C. gilardii genomes.

**TABLE 3:** Susceptibility profile of C. gilardii W2-2 for selected β-lactams, aminoglycosides, and colistin

Antibiotic class and antibiotic	Diam (mm) or MIC (μg/ml), interpretation^*a*^
β-Lactams (subclass)	
Ampicillin (penicillins)	0, R
Amoxicillin-clavulanate (combination)	0, R
Cefotaxime (cephalosporins)	44, S
Ertapenem (carbapenems)	0, R
Imipenem (carbapenems)	23, S
Meropenem (carbapenems)	0, R
Aminoglycosides	
Amikacin	24, S
Gentamicin	0, R
Kanamycin	24, S
Spectinomycin	15, I
Streptomycin	3, R
Tobramycin	10, R
Lipopeptide, colistin	1, S (MIC)

a The diameter (or the MIC for colistin) results shown are averages from at least three independent experiments. Interpretation of resistant (R) or sensitive (S) was performed using the Pseudomonas aeruginosa CLSI zone diameter breakpoint values (or MIC values, for colistin) (63) whenever possible. The *Enterobacteriaceae* breakpoint values were used for those antibiotics for which P. aeruginosa breakpoint values are not provided by CLSI (ampicillin, amoxicillin-clavulanate, cefotaxime, ertapenem, kanamycin, and streptomycin) (63). The amikacin breakpoint values were also used for interpretation of spectinomycin susceptibility, for which no P. aeruginosa or *Enterobacteriaceae* CLSI values are available. I, intermediate.

In contrast, resistance to colistin did not seem to be intrinsic in C. gilardii, despite the recent finding of the *mcr-5* colistin resistance gene in a transposon present in strain CR3 (18). Using comparative genomics and BLAST analysis, we found that neither our water isolate nor strain JZ4 contained this gene. Moreover, when we tested the susceptibility of our isolate to this antibiotic, we found that it was sensitive to colistin (MIC < 0.125 mg/liter; Table 3), as previously found for the only C. gilardii clinical isolate tested for susceptibility to this antibiotic (11).

To identify C. gilardii W2-2 antibiotic resistance genes, we selected for further analysis all 40 genes initially mapped to the Comprehensive Antibiotic Resistance Database (CARD) (19, 20) or the PATRIC AMR database (21), as well as all other genes annotated as antibiotic resistance genes or drug efflux transporters. For each selected gene, we used BLASTP analysis (22, 23) to search the full predicted protein against the proteins in CARD, which is a curated collection of characterized, peer-reviewed antibiotic resistance determinants. The 30 predicted proteins found to have a strong match (generally, greater than 65% similarity and 80% coverage) to one or more antibiotic resistance proteins in CARD are shown in Table S2. Overall, we found that C. gilardii W2-2 has an extensive array of antibiotic resistance genes which are also very well conserved among the other C. gilardii isolates currently sequenced. These resistance genes include genes for a large number of multidrug efflux pumps homologous to major pumps, such as the MexAB-OprM, MexCD-OprJ, MexEF-OprN, and other pumps of P. aeruginosa; the AcrAB-TolC, EmrAB-TolC, and MdtABC-TolC pumps of Escherichia coli; and the AdeFGH pump of Acinetobacter baumannii (Table S2). We also identified genes for putative antibiotic inactivation enzymes for β-lactams (a class D β-lactamase) and aminoglycosides (an aminoglycoside 3-*N*-acetyltransferase and an aminoglycoside 3ʺ-adenylyltransferase) (Table S2). The identification of these multidrug efflux pumps and antibiotic inactivation enzymes provides, for the first time, information about the mechanisms of resistance to multiple antibiotics in C. gilardii.

### Determinants for resistance to β-lactams.

Our genomic analyses revealed a novel chromosomally encoded OXA-like class D β-lactamase (Table S2). We submitted its DNA and protein sequence to the National Center for Biotechnology Information (NCBI) β-Lactamase Alleles database (BioProject accession number PRJNA305729), which curates and assigns names to new β-lactamases. This new β-lactamase has been designated OXA-837 (the gene is designated *bla*_OXA-837_ [GenBank accession number MN313890]). We found this β-lactamase to be well conserved in the C. gilardii CR3 and JZ4 strains (82 to 83% identity). OXA-837 is most closely related (49 to 50% identity, 65% similarity, 77 to 84% coverage) to the intrinsic β-lactamases OXA-60 from Ralstonia pickettii and OXA-50 from P. aeruginosa (24, 25). Such similarity, especially that between OXA-837 and R. pickettii OXA-60, is in agreement with the close relatedness between the genera *Cupriavidus* and *Ralstonia* mentioned in the introduction. We then cloned a codon-optimized version of the *bla*_OXA-837_ gene in E. coli to test its effect on susceptibility to β-lactams (Table 4). We found that OXA-837 is a narrow-spectrum β-lactamase that decreases susceptibility to ampicillin but not the other β-lactams tested in E. coli (Table 4). Such an effect on susceptibility to ampicillin is in agreement with the activity against this antibiotic previously reported for OXA-50 of P. aeruginosa (25) and also with the resistance to this antibiotic found in our C. gilardii isolate (Table 3) and in clinical isolates (11, 16, 17). The fact that OXA-837 had no activity against cefotaxime and imipenem seems to explain why our isolates (Table 3), as well as many clinical isolates (11, 13, 15, 16), are often susceptible to both antibiotics. Resistance to imipenem in some C. gilardii clinical isolates (13, 14, 17) might be related to other mechanisms, such as decreased expression or mutational inactivation of porins, as previously reported for OprD in P. aeruginosa (26–29). However, no information about the expression/mutation of porins in these imipenem-resistant clinical isolates is currently available to confirm this hypothesis. Finally, resistance to meropenem (as well as ertapenem and amoxicillin-clavulanate) was common to both our water isolate (Table 3) and clinical isolates (13–17). OXA-837 did not decrease susceptibility to these antibiotics when cloned in E. coli, which suggests that resistance to these antibiotics in C. gilardii is likely due to other intrinsic mechanisms (Table S2). For example, although the detailed mechanism is not fully understood, *Pseudomonas* species are generally ertapenem resistant because of the low permeability of their outer membrane and their multiple efflux pumps (30, 31), several of which have homologs in C. gilardii (Table S2). Likewise, the MexAB-OprM pump of P. aeruginosa is known to mediate resistance to meropenem but not to imipenem, especially when overexpressed (26, 27, 29). This mechanism may also occur in C. gilardii, given that our isolate was also meropenem resistant and imipenem sensitive and given that all sequenced C. gilardii strains have a very well-conserved homolog of the MexAB-OprM pump (e.g., 79% identity, 89% similarity, and 99% coverage between the MexB RND inner membrane component of strain W2-2 and MexB of P. aeruginosa; Table S2). Future experiments with our strains and with clinical isolates will be necessary to confirm these hypotheses.

**TABLE 4:** Effect of the *bla*_OXA-837_ gene from C. gilardii W2-2 on susceptibility to β-lactam antibiotics in the E. coli host strain

Antibiotic	Diam (mm)^*a*^	
	E. coli DH7298(pBAD18-cm)	E. coli DH7299 (pBAD18-*bla*_OXA-837_)
Ampicillin	26	20*
Amoxicillin-clavulanic acid	24	23
Cefotaxime	40	39
Ertapenem	38	36
Imipenem	33	32
Meropenem	34	34

a The diameter results shown are averages from five independent experiments. *, statistically significant differences (*P* < 0.002) between the E. coli strain with the empty pBAD18-cm plasmid (DH7298) and the strain with the codon-optimized *bla*_OXA-837_ gene from C. gilardii W2-2 cloned into pBAD18-cm (DH7299). Except for ampicillin, all other *P* values were >0.05.

### Aminoglycoside resistance determinants.

We identified two putative aminoglycoside-inactivating genes in strain W2-2 (Table S2). These genes may explain the strong resistance to several drugs of this class found in our water isolate (Table 3) and in clinical isolates (11, 13–17). The first putative aminoglycoside resistance gene that we found in strain W2-2 encodes a novel aminoglycoside 3-*N*-acetyltransferase IV enzyme (Table S2). This aminoglycoside acetyltransferase was well conserved between the other two sequenced C. gilardii isolates ( ≥83% protein identity; Table S2) and may explain the strong resistance to gentamicin and tobramycin found both in our water isolate (Table 3) and in clinical isolates (11, 13–17). Despite the acetyltransferases from strains CR3 and JZ4 being annotated as AAC-VI family aminoglycoside *N*-acetyltransferases, when analyzed against CARD, the aminoglycoside acetyltransferases from these two strains and from our water isolate were most closely related (73% identity, 83% similarity, and 99% coverage for the predicted aminoglycoside acetyltransferase protein from strain W2-2) to a plasmid-encoded gentamicin-3-acetyltransferase, AAC(3)-IVa (AacC4; GenBank accession number ABB43029.1), from E. coli (originally thought to be *Salmonella*) and Pseudomonas stutzeri (32–34) (Table S2). Because of this sequence similarity and because the aminoglycoside acetyltransferase found in our water isolate decreased susceptibility to gentamicin and tobramycin (as expected for a class IV enzyme [35]) when expressed in E. coli (Table 5), we named this novel enzyme found in strain W2-2 AAC(3)-IVb and the gene coding this enzyme *aac(3)-IVb*. There are currently two different nomenclatures used to identify aminoglycoside resistance genes and enzymes (36). In the one used here, “*aac”* (gene) or “AAC” (protein) corresponds to the type of enzymatic activity (aminoglycoside acetyltransferase), “*(3)*” corresponds to the site of modification (class), “*IV*” corresponds to the subclass (activity on gentamicin and tobramycin), and “*b*” distinguishes the new variant identified here from the one originally found in E. coli (32–34). Alternatively, using the second currently used nomenclature, the gene identified in our water isolate would be named *aacC10* (AacC10 for the protein), where “*aac*” corresponds to the type enzymatic activity, “*C*” corresponds to modification of site 3, and “*10*” corresponds to the unique identifier of the gene.

**TABLE 5:** Effect of the aminoglycoside 3-*N*-acetyltransferase *aac(3)-IVb* (*aacC10*) and the aminoglycoside 3ʺ-adenylyltransferase *ant(3ʺ)-Ib* (*aadA32*) genes from C. gilardii W2-2 on susceptibility to aminoglycoside antibiotics in the E. coli host strain

Antibiotic	Diam (mm)^*a*^		
	E. coli DH7285(pUC19)	E. coli DH7284 (pUC19-*aac(3)-IVb*)	E. coli DH7287 (pUC19-*ant(3ʺ)-Ib*)
Amikacin	27	27	26
Gentamicin	26	19**	25
Kanamycin	27	26	26
Spectinomycin	24	24	18**
Streptomycin	21	20	19*
Tobramycin	24	17**	23

a The diameter results shown are averages from five independent experiments. Statistically significant differences between the E. coli strain with the empty pUC19 plasmid (DH7285) and the strains with either the *aac(3*)-*IVb* or *ant(3ʺ)-Ib* gene from C. gilardii W2-2 cloned into pUC19 (strains DH7284 or DH7287, respectively) are indicated: **, *P* < 0.005; *, *P *< 0.05.

Interestingly, the *aac(3)-IVa* gene, first identified in E. coli, is widespread in plasmids of many human clinical and farm animal isolates of E. coli, Klebsiella pneumoniae, Salmonella enterica, and other *Enterobacteriaceae*, generally as a part of genetic mobile elements, such as transposons (37, 38). In contrast, the *aac(3)-IVb* gene of C. gilardii is located on the chromosome, and, based on the lack of nearby insertion sequences, transposase genes, etc., it is not found as part of a genetic mobile element. These differences suggest that the aforementioned *Enterobacteriaceae* clinical/farm isolates might have ultimately acquired their *aac(3)-IVa* gene from C. gilardii or other environmental bacteria in which this gene is intrinsically present. Because of its important clinical implications, we further studied the C. gilardii W2-2 *aac(3)-IVb* gene by cloning it into an E. coli host strain to test its effect on aminoglycoside susceptibility (Table 5). We found that expression of this aminoglycoside 3-*N*-acetyltransferase gene strongly decreases susceptibility to gentamicin and tobramycin in E. coli (Table 5), in agreement with the strong resistance to these two antibiotics found in our C. gilardii isolate (Table 3) and in C. gilardii clinical isolates (11, 13–15, 17). In contrast, this gene had little to no effect on E. coli susceptibility to amikacin and kanamycin, in agreement with the susceptibility to these antibiotics found in our C. gilardii isolate (Tables 3 and 5). In addition, this gene had no significant effect on streptomycin or spectinomycin susceptibility in E. coli, which suggests that the streptomycin-resistant and spectinomycin-intermediate phenotypes found in our isolate (Table 3) may be caused by the second potential aminoglycoside resistance gene identified in strain W2-2.

The second aminoglycoside resistance candidate gene identified in our isolate encodes a protein annotated as an aminoglycoside 3ʺ-adenylyltransferase. We also found a predicted protein 78% identical to the adenylyltransferase from our water isolate in C. gilardii JZ4, whereas we identified no homolog in C. gilardii CR3 (Table S2). The predicted adenylyltransferase of strain W2-2 is most closely related (41% identity, 59% similarity, and 87% coverage) to the integron-encoded aminoglycoside-(3ʺ)(9)-adenylyltransferase AadA16 (GenBank accession number ACF17980.1) found in E. coli and other bacterial clinical isolates (39, 40), as well as to other aminoglycoside adenylyltransferases, such as the integron-encoded aminoglycoside 3ʺ-adenylyltransferase AadA11 (GenBank accession number AAV32840.1) (40% identity, 57% similarity, and 85% coverage) found in P. aeruginosa (41) and other species, such as E. coli (GenBank accession number ACX42431.1) or A. baumannii (GenBank accession number AVF08038.1). According to the two different aforementioned nomenclatures for aminoglycoside resistance determinants, we named the novel enzyme found in our water isolate ANT(3ʺ)-Ib [*ant(3ʺ)-Ib* for the gene], where “ANT” corresponds to the type of enzymatic activity (aminoglycoside adenylyltransferase), “(3ʺ)” corresponds to the site of modification (class), “I” corresponds to the subclass (activity on streptomycin and spectinomycin), and “b” distinguishes the new variant. Using the alternative nomenclature, which is the one most commonly used for adenyltransferases (36), we also named this enzyme AadA32 (*aadA32* for the gene), where “Aad” corresponds to the type enzymatic activity, “A” corresponds to the site of modification, and “32” corresponds to the unique identifier of the gene. As mentioned above, cloning of this gene in E. coli confirmed that C. gilardii W2-2 ANT(3ʺ)-Ib (AadA32) modestly decreases susceptibility to spectinomycin and streptomycin in E. coli (Table 5). These results, combined with the decreased susceptibility to gentamicin and tobramycin observed for AAC(3)-IVb, seem to explain the resistance to different aminoglycosides found both in our water isolate (Table 3) and in clinical isolates (11, 13–17). These enzymes may act synergistically with the vast array of multidrug efflux pumps identified in C. gilardii, including homologs of the P. aeruginosa MexAB-OprM, MexEF-OprN, and MexCD-OrpJ pumps (Table S2), which are known to contribute to aminoglycoside resistance (42).

### Final remarks.

C. gilardii is gaining attention because of its biotechnological potential and role as an emerging multidrug-resistant pathogen. However, most aspects of the biology of this species, including its intrinsic antibiotic resistance mechanisms, remain mostly unknown. Moreover, only two genomes of this species have been sequenced to date. Here, we have studied an environmental C. gilardii isolate which, like the C. gilardii clinical isolates described to date (13–17), is resistant to meropenem, gentamicin, and other β-lactams and aminoglycosides. Biochemical characterization, whole-genome sequencing, and phylogenetic and comparative genomic analyses have confirmed that our isolate is C. gilardii and revealed that it has multiple virulence genes and antibiotic resistance determinants. Further analysis of the genome of this isolate revealed a large intrinsic resistome, well conserved among all three currently sequenced C. gilardii genomes. Such high conservation among isolates from very different environments and the similar antibiotic susceptibility profiles found between our isolate and clinical strains suggest that this resistome may also be conserved in clinical C. gilardii strains. This resistome consists of many multidrug efflux pumps, including a well-conserved homolog of the P. aeruginosa MexAB-OprM pump that may confer decreased susceptibility to meropenem, other β-lactams, and aminoglycosides. This resistome also includes OXA-837, a narrow-spectrum class D β-lactamase that confers decreased susceptibility to ampicillin but not to the other β-lactams tested; a new aminoglycoside 3-*N*-acetyltransferase [AAC(3)-IVb/AacC10] that confers decreased susceptibility to gentamicin and tobramycin; and a novel aminoglycoside 3ʺ-adenylyltransferase [ANT(3ʺ)-Ib/AadA32, absent from strain CR3] that confers decreased susceptibility to spectinomycin and streptomycin. These findings provide the first mechanistic insight into how C. gilardii is intrinsically resistant to multiple antibiotics and how it may become resistant to additional antibiotics during therapy.

## MATERIALS AND METHODS

### Isolation, identification, and characterization of C. gilardii W2-2.

Cupriavidus gilardii strain W2-2 was isolated from a surface-level water sample collected on 2 August 2016 from the CSUN Duck Pond, an artificial urban pond located on the California State University, Northridge, campus (Los Angeles, CA; Global Positioning System location, 34.2367024, −118.5261293). This strain was isolated as part of a wider effort to isolate antibiotic-resistant bacteria from environmental water sources (6). This isolate was identified as C. gilardii by 16S rRNA gene PCR amplification and Sanger sequencing (6).

To phenotypically characterize C. gilardii W2-2, this strain was grown at 37°C overnight on Mueller-Hinton agar plates and then assayed by a combination of morphological, physiological, and biochemical tests that included Gram staining followed by observation at a ×1,000 magnification using bright-field microscopy; the oxidase test, which was performed using the Becton Dickinson BBL DrySlide oxidase reagent (Sparks, MD) as described previously (6); catalase and sulfide indole motility tests, which were performed using standard procedures; and a panel of 20 biochemical tests (see Table S1 in the supplemental material), performed by using API 20NE strips (bioMérieux, Durham, NC) according to the manufacturer’s specifications. E. coli ATCC 25922 was used as a quality control.

### Genome extraction and sequencing of C. gilardii W2-2.

We used a DNeasy blood and tissue kit from Qiagen (Valencia, CA) according to the manufacturer’s specifications to extract the genomic DNA of C. gilardii W2-2. We then assessed the DNA quality using a NanoDrop spectrophotometer (Thermo Fisher Scientific, Canoga Park, CA), the DNA size via gel electrophoresis, and finally, the DNA concentration with a Quant-iT PicoGreen double-stranded DNA (dsDNA) assay kit (Life Technologies, Carlsbad, CA). Next, we prepared a DNA library using a NEBNext Ultra II DNA library preparation kit for Illumina with sample purification beads from New England Biolabs (NEB; Ipswich, MA). DNA was barcoded using NEBNext multiplex oligonucleotides for Illumina (96 index primers; NEB). Before pooling the barcoded sequencing library, we assessed its quality using a Bio-Rad (Hercules, CA) Experion DNA analysis kit and quantified it using the PicoGreen dsDNA assay. Finally, the pooled barcoded library was submitted to GeneWiz (Newbury Park, CA), where the concentration of our pooled library was reconfirmed before sequencing on an Illumina (San Diego, CA) HiSeq X sequencer (paired-end run; 2 × 150 bp). A total of 13,112,726 reads (coverage, ∼351 times) was generated for strain W2-2.

### Genome assembly, annotation, and analysis of C. gilardii W2-2.

Reads for strain W2-2 were submitted to the comprehensive genome analysis service at the Pathosystems Resource Integration Center (PATRIC) (21) for genome assembly using the SPAdes (v3.10.0) genome assembler (43) and genome annotation using genetic code 11 and the RAST tool kit (RASTtk) (44). In addition, the genome assembly was reannotated using the NCBI Prokaryotic Genome Annotation Pipeline (PGAP) (45, 46) during its submission to the GenBank database to ensure full compatibility of the annotations with GenBank standards. The annotation and comprehensive analysis at PATRIC included Enzyme Commission (EC) number assignments according to the BRENDA enzyme database (47), Gene Ontology (GO) assignments (48), and mapping to KEGG pathways (49). It also included assignment to the genus-specific protein families (PLfam) and cross-genus protein families (PGfam) of the microbial genomes PATRIC database (50). Subsystem analysis was used to identify sets of proteins that are involved in the same specific biological process or structural function (51). Mapping to reference genes from external and PATRIC curated databases was used to identify genes involved in antibiotic resistance (CARD [19, 20] and the PATRIC AMR database [21]), virulence (PATRIC_VF [52], Victors [http://www.phidias.us/victors/index.php], and VFDB [53] databases), and transport (TCBD database [54]).

We then further manually investigated all C. gilardii W2-2 antibiotic resistance genes initially mapped to CARD (19, 20) or the PATRIC AMR database (21), as well as all genes that were not initially mapped to CARD or the PATRIC AMR database but that were annotated as potential antibiotic resistance genes or drug efflux transporters. We then used BLASTN/BLASTP analysis (22, 23) to search each full candidate gene/predicted protein against the curated collection of characterized, peer-reviewed antibiotic resistance determinants compiled in CARD. In general, proteins with greater than 80% coverage and 65% similarity to one or more bona fide antibiotic resistance proteins in CARD were considered antibiotic resistance proteins.

### Whole-genome phylogenetic analysis of the genus *Cupriavidus* and comparative genomic analysis of C. gilardii strains.

Phylogenetic analysis of the *Cupriavidus* genus was performed using the phylogenetic tree service at PATRIC (21), including all 61 complete or draft *Cupriavidus* genomes currently available in the PATRIC database (collected from GenBank and other sources) plus the C. gilardii genome obtained in this study. The genome of the type strain Ralstonia solanacearum K60-1 was used as the outgroup. Briefly, the PATRIC phylogenetic tree service uses the PEPR software pipeline and the BLAST (22, 23), MCL (55), MUSCLE (56), hmmbuild (57), hmmsearch (57), Gblocks (58), and RAxML (59) tools to filter and remove duplicate species within the ingroup genome protein files. This process is then followed by BLAST searches to find bidirectional best hit protein pairs between genomes, which are then clustered using the Markov cluster (MCL) algorithm. Clusters containing members from at least half of the distinct genomes are chosen as seed homolog sets, then expanded using the HMMer suite to include members from all ingroup and outgroup taxa, and, finally, used by the hmmbuild tool to build a hidden Markov model (HMM). These HMMs are used to search each genome with the hmmsearch tool, find the best match from each genome for each homolog set model, and create the final homolog sets, which, after removal of those sets representing less than 80% of the ingroup genomes, are aligned using the MUSCLE tool. The alignments were then trimmed using Gblocks, concatenated, and then used to build the phylogenetic tree with the RAxML algorithm and the progressive refinement method, using gene-wise jackknife to estimate branch support values (60).

We then performed a comparative genomics analysis between our C. gilardii isolate and the other two currently available genomes identified to belong to C. gilardii (strain CR3, NCBI GenBank genome accession numbers CP010516 and CP010517; and strain JZ4, GenBank accession number LVXY00000000). First, we used the Mauve (v1.1.1) tool (61) within the Geneious R11 software platform to perform a multiple-genome alignment and generate a synteny plot for all three C. gilardii strains. Next, we used the Proteome Comparison tool at PATRIC (21), which is based on the original Sequence-Based Comparison tool that was part of RAST (62), to determine protein similarity using BLASTP analysis and mark each gene as either unique, a unidirectional best hit, or a bidirectional best hit when it was compared to the reference strain CR3 genome. Finally, we used the generated proteome comparison table to identify which antibiotic resistance genes found in C. gilardii W2-2 were also found among other C. gilardii genomes and to determine the degree of conservation of these genes across all C. gilardii sequenced strains.

### Determination of susceptibility to β-lactams, aminoglycosides, and colistin.

The susceptibility of C. gilardii W2-2 to β-lactams and aminoglycosides was determined as previously described (6), by using the CLSI disk diffusion recommendations (63), disks purchased from Becton, Dickinson (Franklin Lakes, NJ), and E. coli ATCC 25922 as a quality control (63).

To assay susceptibility to colistin (colistin sulfate; Thermo Fisher Scientific), we followed the CLSI recommendations of using a broth microdilution assay instead of a disk diffusion assay (63). The assay was performed with 2-fold serial broth dilutions as previously described (64), using E. coli ATCC 25922 as a quality control (63).

### Cloning of *bla*_OXA-837_ from C. gilardii W2-2 and measuring its effect on susceptibility to β-lactam antibiotics.

To investigate the role of the putative OXA-837 β-lactamase identified in strain W2-2 on resistance to various β-lactam antibiotics, we attempted to amplify and clone this gene in E. coli. However, possibly because of issues with the very high percent GC content of this gene (67% overall, but 75% for the first 115 nucleotides), we were unable to clone and express this construct in E. coli. To address this issue, we designed an E. coli codon-optimized version of this gene with a lower percent GC content (but encoding the same protein) that was synthesized by Integrated DNA Technologies (Coralville, IA). The vector carrying the codon-optimized *bla*_OXA-837_ gene was then used as a template for a PCR performed using Phusion polymerase (Thermo Fisher Scientific); the forward primer 5′-GAGCTC*AGGAGG*AATTCATGAAGAGCCGCACAGAG-3′, which contains a SacI restriction site (underlined) and a Shine-Dalgarno sequence (shown in italics) for subsequent expression in E. coli; and the reverse primer 5′-GTCGACCTAAGACATCTTACGGGCC-3′, which contains a SalI restriction site (underlined). The PCR product was digested with SacI and SalI from NEB and ligated, using the T4 DNA ligase from Thermo Fisher Scientific, into the pBAD18-cm vector (65) that had been digested with the same enzymes, following the manufacturer’s specifications. The plasmid with the *bla*_OXA-837_ gene cloned adjacent to the arabinose-inducible P*_BAD_* promoter was transformed into the E. coli BW25113 host strain (66) to create the E. coli DH7299 strain. The construct in this strain was verified by plasmid extraction and insert sequencing. For control experiments, empty pBAD18-cm was transformed into E. coli BW25113 to create the strain DH7298. The susceptibility of both strains to β-lactam antibiotics was assayed by the disk diffusion method as described above, using Mueller-Hinton (M-H) agar plates containing 0.2% l-arabinose.

### Cloning of C. gilardii W2-2 aminoglycoside 3-*N*-acetyltransferase and aminoglycoside 3ʺ-adenylyltransferase genes and their effect on susceptibility to aminoglycosides.

To investigate the contribution of the aminoglycoside 3-*N*-acetyltransferase *aac(3)-IVb* (*aacC10*) and aminoglycoside 3ʺ-adenylyltransferase *ant(3ʺ)-Ib* (*aadA32*) genes from C. gilardii W2-2 to aminoglycoside resistance, we cloned both genes under the control of an IPTG (isopropyl-β-d-thiogalactopyranoside)-inducible *lac* promoter and exogenously expressed it in an E. coli host. To clone these genes, we first amplified each gene by PCR using DreamTaq polymerase from Thermo Fisher Scientific, the forward primer 5′-GATCGGTACCATGTTGGTGACCCAGTTG-3′ [for *aac(3)-IVb*] or 5′-GATCGGATCCATGCCACCGCCTG-3′ [for *ant(3ʺ)-Ib*] (the KpnI restriction site is underlined), and the reverse primer 5′-GATCGAATTCCTACTTGGTGCTGACC-3′ [for *aac(3)-IVb*] or 5′-GATCGAATTCTCATGCCTTGACGCT-3′ [for *ant(3ʺ)-Ib*] (the EcoRI restriction site is underlined). The amplified *aac(3)-IVb* and *ant(3ʺ)-Ib* genes were digested with KpnI and EcoRI from NEB and ligated using the T4 DNA ligase from Thermo Fisher Scientific into the pUC19 plasmid (67) that had been digested with the same enzymes to generate the pUC19-*aac(3)-IVb* and pUC19-*ant(3ʺ)-Ib* constructs, respectively, following the manufacturer’s specifications. The constructs were transformed into the E. coli BW25113 (66) host strain to create the strains DH7284 and DH7287, respectively. The constructs in these strains were verified by plasmid extraction and insert sequencing. For control experiments, empty pUC19 was transformed into BW25113 to create the strain DH7285. The susceptibility of all three strains to aminoglycoside antibiotics was assayed by the disk diffusion method as described above, using M-H agar plates containing 100 μM IPTG.

### Data availability.

The sequence of the C. gilardii W2-2 *bla*_OXA-837_ β-lactamase gene has been made public by depositing it in the National Center for Biotechnology Information (NCBI) GenBank database (GenBank accession number MN313890) and the NCBI β-Lactamase Alleles database (BioProject accession number PRJNA305729). The sequences of the C. gilardii W2-2 *aac(* 3*)-IVb* (*aacC10*) aminoglycoside 3-*N*-acetyltransferase gene and the *ant(3ʺ)-Ib* (*aadA32*) aminoglycoside 3ʺ-adenylyltransferase gene have been deposited in GenBank under accession numbers MN366378 and MN366379, respectively. The Whole Genome Shotgun project of C. gilardii W2-2 has been deposited in DDBJ/ENA/GenBank under accession number VSRI00000000. The version described in this paper is the version with GenBank accession number VSRI01000000.
