# Supplementary material for: Comparative Genomics Reveals a Well-Conserved Intrinsic Resistome in the Emerging Multidrug-Resistant Pathogen Cupriavidus gilardii
Source: mSphere. 2019 Oct 2;4(5):e00631-19. doi: 10.1128/mSphere.00631-19 (PMC6796972; doi:10.1128/mSphere.00631-19)
Supplement: TABLE S1 [file mSphere.00631-19-st001.pdf]

TABLE S1 Phenotypic characterization of *C. gilardii* W2-2.

**Test**	**Reaction/enzyme**	***C. gilardii* W2-2**
Colonies	Morphology of colonies grown overnight at 37°C on Mueller-Hinton agar	circular, 0.5-1 mm in diameter, white-yellowish
Gram stain	Gram stain and cell morphology	Gram-negative rods
Catalase	Catalase	+
SIM Motility	Bacterial motility in 0.4% agar	+
**API 20NE**		
NO3	Reduction of nitrate to nitrites/nitrogen	-
TRP	Indole production	-
GLU_Ferm	Fermentation of glucose	-
ADH (Arg)	Arginine Dihydrolase	-
URE	Urease	-
ESC	Esculin hydrolysis (β-glucosidase)	-
GEL	Gelatin hydrolysis	-
PNPG	β-galactosidase	-
GLU_Assim	Assimilation of glucose	-
ARA	Assimilation of arabinose	-
MNE	Assimilation of mannose	-
MAN	Assimilation of mannitol	-
NAG	Assimilation of N-acetyl-glucosamine	-
MAL	Assimilation of maltose	-
GNT	Assimilation of potassium gluconate	+
CAP	Assimilation of capric acid	+
ADI	Assimilation of adipic acid	+
MLT	Assimilation of malate	+
CIT	Assimilation of trisodium citrate	-
PAC	Assimilation of phenylacetic acid	-
OX	Cytochrome c oxidase	+
API ID		3822
