# Supplementary material for: Comparative Genomics Reveals a Well-Conserved Intrinsic Resistome in the Emerging Multidrug-Resistant Pathogen Cupriavidus gilardii
Source: mSphere. 2019 Oct 2;4(5):e00631-19. doi: 10.1128/mSphere.00631-19 (PMC6796972; doi:10.1128/mSphere.00631-19)
Supplement: TABLE S2 [file mSphere.00631-19-st002.pdf]

TABLE S2 Antibiotic resistance determinants found in *C. gilardii* W2-2 with a strong[a] match in the CARD database.

Locus tag (GenBank)	Predicted protein in *C. gilardii* W2-2 (PATRIC annotation)	Closest match in CARD database[b]				% Identity to W2-2[c]	
		Closest match in CARD database	% Identity	% Similarity	% Coverage	CR3	JZ4
FX016_22760 (MN313890)	Class D beta-lactamase OXA-837 (EC 3.5.2.6)	*Ralstonia picketti* OXA-60 β-lactamase<br>*P. aeruginosa* OXA-50 β-lactamase	49<br>50	65<br>65	84<br>77	83	82
FX016_08315 (MN366378)	Aminoglycoside 3-N-acetyltransferase [AAC(3)-IVb / AacC10] (EC 2.3.1.81)	*Escherichia coli* and *Pseudomonas stutzeri* AAC(3)-IVa	73	83	99	83	83
FX016_04685 (MN366379)	Aminoglycoside 3''-adenylyltransferase [ANT(3'')-Ib / AadA32] (EC 2.7.7.47)	*E. coli* aminoglycoside-(3'')(9)-adenylyltransferase AadA16<br>*Pseudomonas aeruginosa* streptomycin 3''-adenylyltransferase AadA11	41<br>40	59<br>57	87<br>85	Absent	78
FX016_17790	Multidrug efflux system EmrAB-OMF, inner-membrane proton/drug antiporter EmrB (MFS type)	EmrB component of *E. coli* EmrAB-TolC pump	55	71	93	98	95
FX016_17785	Multidrug efflux system EmrAB-OMF, membrane fusion component EmrA	EmrA component of *E. coli* EmrAB-TolC pump	47	64	91	99	95
FX016_17780	Outer membrane factor (OMF) lipoprotein associated with EmrAB-OMF efflux system	AdeH component of *Acinetobacter baumannii* AdeFGH pump	35	54	80	96	95
FX016_05295	Multidrug efflux system MdtABC-TolC, inner-membrane proton/drug antiporter MdtB (RND type)	MdtB component of *E. coli* MdtABC-TolC pump<br>MuxB component of *P. aeruginosa* MuxABC-OpmB pump	68<br>72	82<br>84	97<br>98	99	99
FX016_05290	Multidrug efflux system MdtABC-TolC, inner-membrane proton/drug antiporter MdtC (RND type)	MdtC component of *E. coli* MdtABC-TolC pump<br>MuxC component of *P. aeruginosa* MuxABC-OpmB pump	60<br>59	74<br>73	98<br>99	98	99

FX016_ 05300	Multidrug efflux system MdtABC-TolC, membrane fusion component MdtA	MdtA component of *E. coli* MdtABC-TolC pump<br>MuxA component of *P. aeruginosa* MuxABC-OpmB pump	46<br>53	63<br>71	73<br>62	96	97
FX016_ 05285	Outer membrane factor (OMF) lipoprotein associated with MdtABC efflux system	OpmB component of *P. aeruginosa* MuxABC-OpmB pump	53	67	91	94	89
FX016_ 08510	RND efflux system, inner membrane transporter	AcrB component of *E. coli* AcrAB-TolC pump	61	77	97	99	99
FX016_ 08515	RND efflux system, membrane fusion protein	AcrA component of *E. coli* AcrAB-TolC pump	49	67	94	97	97
FX016_ 08505	Efflux transport system, outer membrane factor (OMF) lipoprotein	OprM component of *P. aeruginosa* MexAB-OprM and other pumps	49	67	96	96	96
FX016_ 02620	RND efflux system, inner membrane transporter	MexB component of *P. aeruginosa* MexAB-OprM pump	79	89	99	97[d]	97
FX016_ 02625	Multidrug efflux system, membrane fusion component MexA of MexAB-OprM	MexA component of *P. aeruginosa* MexAB-OprM pump	65	82	98	95[d]	95
FX016_ 00070	Multidrug efflux system, inner membrane proton/drug antiporter (RND type) MexD of MexCD-OprJ system	MexD component of *P. aeruginosa* MexCD-OprJ pump	67	79	98	90	92
FX016_ 00075	Multidrug efflux system, membrane fusion component MexC of MexCD-OprJ system	MexC component of *P. aeruginosa* MexCD-OprJ pump	62	75	92	97	97
FX016_ 00065	Efflux transport system, outer membrane factor (OMF) lipoprotein	OprJ component of *P. aeruginosa* MexCD-OprJ pump	60	71	91	91	92
FX016_ 03005	RND efflux system, inner membrane transporter	AdeF component of *Acinetobacter baumannii* AdeFGH pump	76	86	99	99	98
FX016_ 03010	RND efflux system, membrane fusion protein	AdeG component of *Acinetobacter baumannii* AdeFGH pump	55	73	88	98	98
FX016_ 03000	Efflux transport system, outer membrane factor (OMF) lipoprotein	AdeH component of *Acinetobacter baumannii* AdeFGH pump	51	68	93	98	98
FX016_ 08590	RND efflux system, inner membrane transporter	MuxB component of *P. aeruginosa* MuxABC-OpmB pump	47	66	98	98	98

FX016_ 08595	RND efflux system, membrane fusion protein	MuxA component of *P. aeruginosa* MuxABC-OpmB pump	36	55	92	98	98
FX016_ 08585	Hypothetical protein	MdsC component of *Salmonella enterica* MdsABC pump	30	49	24	85	90
FX016_ 13945	RND efflux system, inner membrane transporter	MdsB component of *Salmonella enterica* MdsABC pump<br>MexF component of *P. aeruginosa* MexEF-OprN pump	46<br>46	65<br>64	99<br>99	40	95
FX016_ 13950	RND efflux system, membrane fusion protein	MdsA component of *S. enterica* MdsABC pump<br>MexE component of *P. aeruginosa* MexEF-OprN pump	38<br>34	57<br>52	77<br>89	36	87
FX016_ 13940	Efflux transport system, outer membrane factor (OMF) lipoprotein	OprN component of *P. aeruginosa* MeEF-OprN pump<br>OpmD component of *P. aeruginosa* MexGHI-OpmD pump	31<br>33	48<br>50	92<br>95	34	87
FX016_ 16430	RND efflux system, inner membrane transporter	MexK component of *P. aeruginosa* MexJK-OprM/H pump	54	70	99	99	99
FX016_ 16435	CzcABC family efflux RND transporter, membrane fusion protein	MexJ component of *P. aeruginosa* MexJK-OprM/H pump	40	56	71	93	95
FX016_ 08485	Fosmidomycin resistance protein	RosA component of *Yersinia enterocolitica* RosAB pump	60	75	96	95	98

[a]Table S2 includes the 30 predicted proteins from *C. gilardii* W2-2 found to have a strong match in the CARD database. We identified these predicted proteins by analyzing all 40 genes initially mapped to the CARD or PATRIC AMR databases (See Material and Methods in the manuscript for full details), as well as all other genes annotated as potential antibiotic resistance genes or drug efflux transporters. In general, predicted proteins were considered to have a strong match in the CARD database when they had greater than 65% similarity and 80% coverage to one or more antibiotic resistance proteins in the CARD database. Despite being below the aforementioned threshold, we also included ANT(3")-Ib (AadA32), because of its annotation as a aminoglycoside 3"-adenylyltransferase, and because it decreased susceptibility to

spectinomycin and streptomycin when cloned in *E. coli* (see Table 4 in the manuscript). For multicomponent efflux pumps that appear to be encoded by genes in the same operon, all components found in *C. gilardii* W2-2 are shown in the table if at least one of them met the aforementioned threshold. Predicted proteins are designated using their PATRIC annotation, which provides more information regarding protein function; however, the corresponding GenBank locus tag identifier is also provided. For the first 3 proteins in the table, which were cloned and their effect on antibiotic susceptibility tested (Tables 4 and 5), their official annotation is used and both their locus tag identifier in the genome, as well as their individual accession number (shown in parenthesis), are provided.

[b]Closest CARD match for the predicted protein in *C. gilardii* W2-2. The percent (%) identity, similarity and coverage between the predicted protein of *C. gilardii* W2-2 its closest CARD match are also shown.

[c]Percent identity between the predicted protein of *C. gilardii* W2-2 and the predicted proteins of *C. gilardii* CR3 or JZ4 strains.

[d]MexA and MexB in *C. gilardii* CR3 are annotated together as a single predicted protein labeled MexB.

Table S2 footnote, continued.
