# Supplementary figures and images for: Comparative Genomics Reveals a Well-Conserved Intrinsic Resistome in the Emerging Multidrug-Resistant Pathogen Cupriavidus gilardii
Source: mSphere. 2019 Oct 2;4(5):e00631-19. doi: 10.1128/mSphere.00631-19 (PMC6796972; doi:10.1128/mSphere.00631-19)

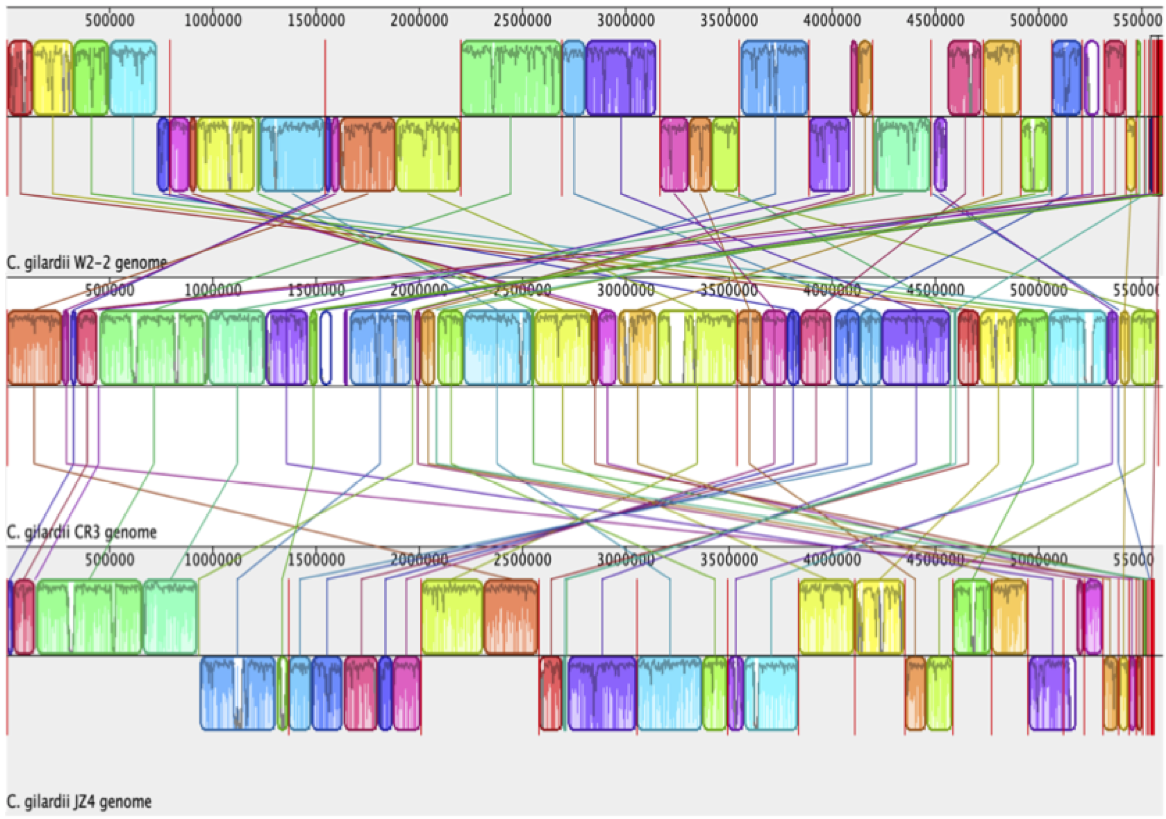

Supplement: FIG S1 [file mSphere.00631-19-sf001.tif]
